# Regional Disconnection in Alzheimer Dementia and Amyloid-Positive Mild Cognitive Impairment: Association Between EEG Functional Connectivity and Brain Glucose Metabolism

**DOI:** 10.1089/brain.2020.0785

**Published:** 2020-12-14

**Authors:** Una Smailovic, Thomas Koenig, Irina Savitcheva, Konstantinos Chiotis, Agneta Nordberg, Kaj Blennow, Bengt Winblad, Vesna Jelic

**Affiliations:** ^1^ Division of Clinical Geriatrics, Center for Alzheimer Research, Department of Neurobiology, Care Sciences and Society, Karolinska Institutet, Stockholm, Sweden.; ^2^ Translational Research Center, University Hospital of Psychiatry and Psychotherapy, University of Bern, Bern, Switzerland.; ^3^ Medical Radiation Physics and Nuclear Medicine, Karolinska University Hospital, Stockholm, Sweden.; ^4^ Department of Neurology, Karolinska University Hospital, Stockholm, Sweden.; ^5^ Clinic for Cognitive Disorders, Karolinska University Hospital-Huddinge, Huddinge, Sweden.; ^6^ Clinical Neurochemistry Laboratory, Department of Psychiatry and Neurochemistry and Sahlgrenska University Hospital, Institute of Neuroscience and Physiology, The Sahlgrenska Academy at the University of Gothenburg, Mölndal, Sweden.; ^7^ Division of Neurogeriatrics, Center for Alzheimer Research, Department of Neurobiology, Care Sciences and Society, Karolinska Institutet, Stockholm, Sweden.; ^8^ Department of Geriatrics, Karolinska University Hospital, Huddinge, Sweden.

**Keywords:** Alzheimer's disease, electroencephalography, [^18^F]fluorodeoxyglucose-PET, functional connectivity, standardized low-resolution electromagnetic tomography (sLORETA)

## Abstract

***Introduction:*** The disconnection hypothesis of Alzheimer's disease (AD) is supported by growing neuroimaging and neurophysiological evidence of altered brain functional connectivity in cognitively impaired individuals. Brain functional modalities such as [^18^F]fluorodeoxyglucose positron-emission tomography ([^18^F]FDG-PET) and electroencephalography (EEG) measure different aspects of synaptic functioning, and can contribute to understanding brain connectivity disruptions in AD.

***Aim:*** This study investigated the relationship between cortical glucose metabolism and topographical EEG measures of brain functional connectivity in subjects along AD continuum.

***Methods:*** Patients diagnosed with mild cognitive impairment (MCI) and AD (*n* = 67), and stratified into amyloid-positive (*n* = 32) and negative (*n* = 10) groups according to cerebrospinal fluid Aβ42/40 ratio, were assessed with [^18^F]FDG-PET and resting-state EEG recordings. EEG-based neuroimaging analysis involved standardized low-resolution electromagnetic tomography (sLORETA), which estimates functional connectivity from cortical sources of electrical activity in a 3D head model.

***Results:*** Glucose hypometabolism in temporoparietal lobes was significantly associated with altered EEG functional connectivity of the same regions of interest in clinically diagnosed MCI and AD patients and in patients with biomarker-verified AD pathology. The correlative pattern of disrupted connectivity in temporoparietal lobes, as detected by EEG sLORETA analysis, included decreased instantaneous linear connectivity in fast frequencies and increased lagged linear connectivity in slow frequencies in relation to the activity of remaining cortex.

***Conclusions:*** Topographical EEG measures of functional connectivity detect regional dysfunction of AD-vulnerable brain areas as evidenced by association and spatial overlap with the cortical glucose hypometabolism in MCI and AD patients.

**Impact statement:**

The association between glucose hypometabolism, as evidenced by [^18^F]FDG-PET ([^18^F]fluorodeoxyglucose positron-emission tomography), and altered electroencephalography (EEG) functional connectivity metrics within temporoparietal lobes provides link between synaptic, neurophysiological, and metabolic impairment in mild cognitive impairment and Alzheimer's disease patients. This study reported alterations in EEG measures of both instantaneous and lagged linear connectivity across distinct frequency bands, both of which were shown to be important for inter- and intrahemispheric communication and function of memory systems in general. EEG-based imaging of brain functional connectivity has a potential to serve as a noninvasive, low-cost, and widely available alternative in assessing synaptic and network dysfunction in cognitively impaired patients.

## Introduction

The disconnection hypothesis of Alzheimer's disease (AD) has emerged as a result of the growing neuroimaging and neurophysiological evidence of altered brain functional connectivity in cognitively impaired individuals (Delbeuck et al., 2003). This notion has been substantiated by neuropathological findings in AD patients, including widespread propagation of AD pathological hallmarks, such as amyloid-beta (Aβ) and tau deposits, damage and loss of synapses, and large corticocortical connections in the brain (Corder et al., 2000; Delbeucket al., 2003; Uylings and de Brabander, 2002). Other evidence that supports this hypothesis includes initial accumulation of Aβ, the main culprit of the disease according to the prevailing amyloid hypothesis (Selkoe and Hardy, 2016), in regions that exhibit changes in brain connectivity in preclinical AD as evidenced by functional magnetic resonance imaging (fMRI) (Palmqvist et al., 2017). These findings encourage the investigation of disturbances in resting-state brain connectivity as a potential biomarker of AD. In addition, AD develops on a clinical and biological continuum, including clinically asymptomatic individuals, subjects with subjective impairment, patients with mild cognitive impairment (MCI) and AD dementia, and is characterized by a typical cascade of dynamic biomarker changes (Sperling et al., 2011). However, neuropathological and neuroimaging markers, the latter including both conventional and novel molecular imaging methods, pose either delayed (postmortem) or costly diagnostic alternatives.

High temporal resolution of the electroencephalography (EEG) technique, which directly measures brain synaptic activity, makes it convenient for investigating synchrony of the underlying large-scale neuronal populations. Numerous studies have already reported disturbances in EEG functional connectivity early during the course of AD, supporting the hypothesis of a disconnection syndrome. The most consistent findings that aroused from the global scalp EEG signal analysis include decreased both instantaneous (Koenig et al., 2005; Smailovic et al., 2018) and lagged connectivity measures (Stam et al., 2007) in fast frequency bands in patients along AD continuum. Referred disturbances in the EEG connectivity measures were associated with the degree of cognitive impairment and molecular markers of AD in the cerebrospinal fluid (CSF) (Engels et al., 2015; Ma et al., 2014; Smailovic et al., 2018). Even though these global scalp EEG measures summarize substantial amount of data, have straightforward computation and a user-friendly interpretation, they lack localization properties.

Functional imaging techniques such as standardized low-resolution electromagnetic tomography (sLORETA), on the contrary, estimate 3D distribution of cortical sources of EEG rhythms in a human head model and can be used for investigation of regional disturbances in brain functional connectivity (Pascual-Marqui et al., 1994). Electrical neuroimaging is therefore a potential technique for early and longitudinal assessment of changes in brain connectome during AD pathogenesis.

In addition to EEG and fMRI, resting-state network dysfunction has been proposed by [^18^F]fluorodeoxyglucose positron-emission tomography ([^18^F]FDG-PET) imaging in MCI and AD patients (Pagani et al., 2017a; Toussaint et al., 2012). However, in the clinical settings, [^18^F]FDG-PET is mainly used to estimate topographical distribution of resting-state glucose utilization in the brain. Decreased glucose metabolism in the temporoparietal regions is the most consistent [^18^F]FDG-PET finding and a supportive marker of possible AD (Mosconi, 2005). Previous studies have shown that the referred pattern of glucose hypometabolism correlates with the degree of cognitive deficits and is predictive of future transition to AD dementia in patients with MCI (Chetelat et al., 2003; Desgranges et al., 1998; Herholz et al., 2002; Pagani et al., 2017b).

Both EEG and [^18^F]FDG-PET are functional modalities that provide information on synaptic activity, by mirroring direct summation of excitatory and inhibitory postsynaptic potentials and indirect metabolic demands of the neurons, respectively. However, studies investigating complementarity of these two modalities as topographical markers of neuronal and synaptic dysfunction, the latter being an early event and best correlate of cognitive deficits in AD patients (Terry et al., 1991), are still scarce. A correlation between the spatial indices of brain glucose metabolism and the localization of the brain electrical center of gravity has been reported (Dierks et al., 2000), as well as an association between the index of cortical glucose hypometabolism and EEG delta activity when analyzed in the same regions of interest (ROIs), comprised of frontal association, ventromedial frontal, temporoparietal, posterior cingulate, and precuneus areas, as assessed by EEG LORETA analysis (Babiloni et al., 2016). Nevertheless, relationship between the regional glucose hypometabolism and the topography of altered brain functional connectivity evidenced by EEG is yet to be investigated in the context of AD.

The main aim of this study was to investigate whether EEG measures of functional connectivity may provide similar information as [^18^F]FDG-PET on selective regional dysfunction of vulnerable brain areas in patients along AD continuum and with biomarker-verified AD molecular pathology. In contrast to the prior approaches, EEG functional connectivity metrics employed in this study were estimated by sLORETA, and included measures of both instantaneous and lagged linear connectivity in four conventional frequency bands. We hypothesized that both measures might provide comprehensive information on regional cortical disconnection associated with AD-related processes such as decreased glucose metabolism in temporoparietal lobes.

## Methods

### Study population

The study included 67 memory clinic patients clinically diagnosed with MCI (*n* = 41) and AD (*n* = 26). All patients underwent comprehensive clinical assessment starting with physical and neurological examination and neuropsychological testing, and were recruited at Karolinska University Hospital Huddinge, Sweden. The clinical diagnoses of MCI or AD were established according to the Winblad and colleagues (2004) and ICD-10 criteria (World Health Organization, 1992), respectively. At the following consensus diagnostic round, results of computed tomography (CT) or MRI brain imaging and conventional CSF biomarker analysis (where available) were taken into account when establishing final diagnosis. [^18^F]FDG-PET imaging and resting-state EEG recordings were additionally performed to exclude other possible differential diagnoses.

This study evaluated, as a first part, the relationship between EEG and [^18^F]FDG-PET in a sample of initially clinically diagnosed MCI and AD patients. As a second step, the relationship between the two modalities was determined in a subsample of patients with biomarker confirmed amyloid pathology.

For the second part of the study analysis, 42 patients (24 MCI and 18 AD) who underwent lumbar puncture and had frozen CSF available from the biobank were stratified according to their amyloid status. For the purpose of this study, CSF Aβ42/40 ratio was additionally analyzed as it compensates for between-individual differences in “total” CSF Aβ (using Aβ40 as a proxy), and it was shown to have a better diagnostic performance than Aβ42 measurement alone (Hansson et al., 2007; Lewczuk et al., 2015). Based on their amyloid status (cutoff for a positive CSF Aβ42/40 ratio <0.89), 14 of 24 MCI were reclassified as amyloid-positive MCI or “MCI due to AD.” Ten MCI subjects were amyloid negative and were not included in the second correlative [^18^F]FDG-PET–EEG analysis. All of the clinically diagnosed AD patients with available CSF sample (*n* = 18) were amyloid positive according to the CSF Aβ42/40 ratio. In total, 32 amyloid-positive, including “MCI due to AD” (*n* = 14) and AD (*n* = 18), patients were included in the second part of the study analyses.

Demographics together with MMSE data in clinical MCI and AD and amyloid-positive and negative patient groups are summarized in Table 1. The exclusion criteria included presence of major psychiatric or neurological comorbidity, epilepsy, psychotropic medication with effect on EEG, alcohol abuse, and time gap between [^18^F]FDG-PET and EEG recording >6 months.

**Table 1.** Demographic and Clinical Characteristics of the Study Population

	Clinical diagnosis (*n* = 67)			Amyloid status (*n* = 42/67)		
	MCI	AD	p^a^	Amyloid negative	Amyloid positive	p^a^
*N*	41	26		10 (all MCI)	32 (MCI+AD)	
Age, years	68 (46–82)	63 (48–86)	0.403	63.5 (46–82)	67 (48–86)	0.652
Sex ratio, males/females	20/21	14/12	0.686	8/2	15/17	0.066
Education, years	13 (7–19)	14 (5–22)	0.766	14.5 (10–18)	11.5 (5–19)	0.202
MMSE	28 (23–30)	23.5 (7–30)	<0.001	27 (24–30)	25 (7–30)	0.286

Data are presented as medians and full ranges (minimum–maximum). Amyloid status was based on CSF Aβ42/20 ratio (the cutoff for CSF Aβ42/40 ratio positivity was <0.89).

^a^ *p*-Values from the Mann–Whitney *U* test or chi-square as appropriate.

AD, Alzheimer's disease; CSF, cerebrospinal fluid; MCI, mild cognitive impairment; MMSE, minimental state examination.

The study was conducted in accordance with Declaration of Helsinki, and written informed consent was obtained from all participants. This study was approved by the Local ethical committee of the Karolinska Hospital and Regional Ethical Review Board in Stockholm (Dnr: 2011/1978-31/4, 2020-00678).

### CSF sampling and analysis

The CSF was obtained from a subset of patients by a routine lumbar puncture procedure (between the L3/L4 or L4/L5 intervertebral space) using a 25-gauge needle and collected in 12 mL polypropylene tubes. The samples were then centrifuged at 1000 rpm for 10 min, divided into 1 mL aliquots, and frozen at −70°C. CSF Aβ42/40 ratio was analyzed using Meso Scale Discovery electrochemiluminescence assay (Vplex; MSD). The cutoff for CSF Aβ42/40 ratio positivity was <0.89.

### [^18^F]FDG-PET data acquisition and analysis

All patients underwent PET/CT scan (Biograph mCT Siemens, Knoxville, TN) as part of the clinical assessment at the Division of Nuclear Medicine, Karolinska University Hospital Huddinge. [^18^F]FDG-PET is performed as a 10–15 min scan 30–45 min after intravenous injection of 2–3 MBq/kg weight. PET acquisition was performed with all appropriate corrections as described previously in detail (Leuzy et al., 2019).

Images was viewed and processed using Syngo.via program (Siemens, Germany). The data were extracted using “MI Neurology” workflow, including automatic template-based spatial normalization and calculation of mean [^18^F]FDG standardized uptake value ([^18^F]FDG-SUV) for 11 ROIs (10 composite cortical ROIs and the whole cerebellum ROI). Cortical ROIs were merged to correspond to bilateral five main brain lobes (frontal, parietal, occipital, temporal, and limbic lobe, which corresponded to the mesial temporal lobe in Syngo software). ROIs were licensed from CEA/Groupe d'Imagerie Fonctionnelle as published in automated anatomical labeling atlas by Tzourio-Mazoyer and colleagues (2002). [^18^F]FDG SUV ratios (SUVRs) were calculated for each of 10 cortical ROIs using the whole cerebellum as a reference region to provide an overview of differences in brain glucose metabolism in MCI and AD groups. In accordance with the hypothesis of this study, the two most vulnerable [^18^F]FDG-PET ROIs (bilateral parietal and temporal lobes) were further correlated with EEG functional connectivity measures in the corresponding brain regions.

### EEG recordings and preprocessing

All patients underwent resting-state eyes-closed EEG recording on the Nervus System (NicoletOne EEG Reader v5.93.0.424; Natus NicoletOne, Pleasanton, CA) at the Department of Clinical Neurophysiology, Karolinska University Hospital Huddinge. Nineteen scalp electrodes were placed according to the standard 10/20 system. The patients' vigilant states were monitored by a technician during the whole EEG recording. The sampling rate of the EEG recording was 256 Hz with the band-pass filters between 0.5 and 70 Hz and electrode impedance <5 kΩ. The recording setup was described previously in detail by Smailovic and colleagues (2018).

EEG data were preprocessed using (i) independent component analysis algorithm for removal of ocular artifacts; and (ii) visual inspection and manual artifact rejection for removal of remaining artifacts and periods of eyes open and nonresting-state vigilant states in Brain Vision Analyzer, version 2.0 software (Gilching, Germany).

### EEG sLORETA analysis

Preprocessed EEG data were further analyzed using sLORETA, which is one of the linear inverse solution techniques that estimates 3D distribution of cortical sources of neuronal activity that give rise to the scalp EEG data (Pascual-Marqui, 2002, 2007a). sLORETA was shown to have improved performance compared with alternative linear inverse algorithms (Pascual-Marqui, 1999) and has been previously validated through studies that combined LORETA and structural and fMRI, [^18^F]FDG-PET as well as intracranial electrode recordings (Dierks et al., 2000; Mulert et al., 2004; Worrell et al., 2000; Zumsteg et al., 2006).

The sLORETA solution space is restricted to the MNI152 template that includes 6239 cortical gray matter voxels at the resolution of 5 mm (Mazziotta et al., 2001). The MNI152 template contains MNI coordinates with neuroanatomical labels based on the “corrected” Talairach coordinates (Brett et al., 2002). The current sources estimated by sLORETA can be averaged across all voxels in a given ROI. In this study, sLORETA ROI transformation matrix included five (bilateral) main brain lobes (frontal, parietal, temporal, occipital, and limbic) that were matched to the corresponding PET ROIs (Tzourio-Mazoyer et al., 2002), with an exception of limbic lobe that did not include hippocampus. The solution space was restricted to the cortical gray matter since it is debatable whether the activity of deep brain structures, such as hippocampus, can be correctly recorded and visualized by sLORETA in the context of correlative PET-EEG imaging study.

sLORETA is a functional imaging technique that can be used to study brain functional connectivity between pairs of ROI. In more detail, the tool named instantaneous linear connectivity, as implemented in LORETA software, measures statistical zero-lag linear dependence between cortical source activities at a given frequency (Pascual-Marqui, 2007b). It is important to note that EEG measures of instantaneous brain connectivity should be interpreted with caution since they are confounded by volume conduction effect, that is, zero-lag spread of the electric fields from their source through the tissue that leads to the nonphysiological inflation of the instantaneous connectivity measures. Still, these measures should not be completely disregarded since physiological zero time-lag synchronization was shown to be important for integrated and coordinated neuronal communication between different brain regions (Fell and Axmacher, 2011; Gollo et al., 2011; Roelfsema et al., 1997). The second measure from LORETA toolbox, named lagged linear connectivity, measures the statistical linear dependence between cortical source activities after the instantaneous zero-lag contribution has been removed, and therefore eliminates volume conduction effect (Pascual-Marqui, 2007b; Pascual-Marqui et al., 2011). Lagged linear connectivity measure, as estimated by LORETA software, has been successfully employed in several EEG studies on functional cortical connectivity in AD patients (Babiloni et al., 2018a,b; Foresta et al., 2019).

In line with our hypothesis and their complementary physiological implications, measures of both instantaneous and lagged linear connectivity (Pascual-Marqui, 2007b) were computed separately between each pair of (bilateral) temporal and parietal lobe and all the remaining ROIs (main brain lobes). Next, average instantaneous and lagged linear connectivity were computed between each bilateral parietal and temporal lobes and all of the remaining nine brain lobes. This resulted in a measure of average instantaneous and lagged linear connectivity of parietal and temporal lobes in relation to the activity of the remaining cortex. Both instantaneous and lagged linear connectivity measures were computed in the conventional frequency bands, including delta (1–3.5 Hz), theta (4–7.5 Hz), alpha (8–11.5 Hz), and beta (12–19.5 Hz) bands.

### Statistical analysis

Statistical analysis was performed in SPSS (version 26; IBM, NY). Nonparametric tests were used in all analyses since the data of interest were not normally distributed. Demographics, MMSE, and [^18^F]FDG SUVRs were compared between the groups using the Mann–Whitney *U* and chi-square tests. The associations between brain glucose metabolism ([^18^F]FDG SUVR) and EEG sLORETA instantaneous and lagged linear connectivity measures in temporoparietal regions were investigated using Spearman's rank correlation tests. Since the analysis was exploratory, the results were presented with both uncorrected and corrected *p*-values (Benjamini–Hochberg correction with 5% false discovery rate) for multiple comparisons. Significance threshold value was *p* < 0.05 for all statistical tests.

### Data availability statement

The datasets used and/or analyzed during this study are available from the study's senior and corresponding authors on reasonable request.

## Results

### Demographics

Demographic data and MMSE scores in clinically diagnosed MCI and AD patients and in amyloid-positive and negative patient groups are presented in Table 1. There were no statistically significant differences in age, sex, or education between the groups.

### Brain glucose metabolism in MCI and AD patients

**FIG. 1.**
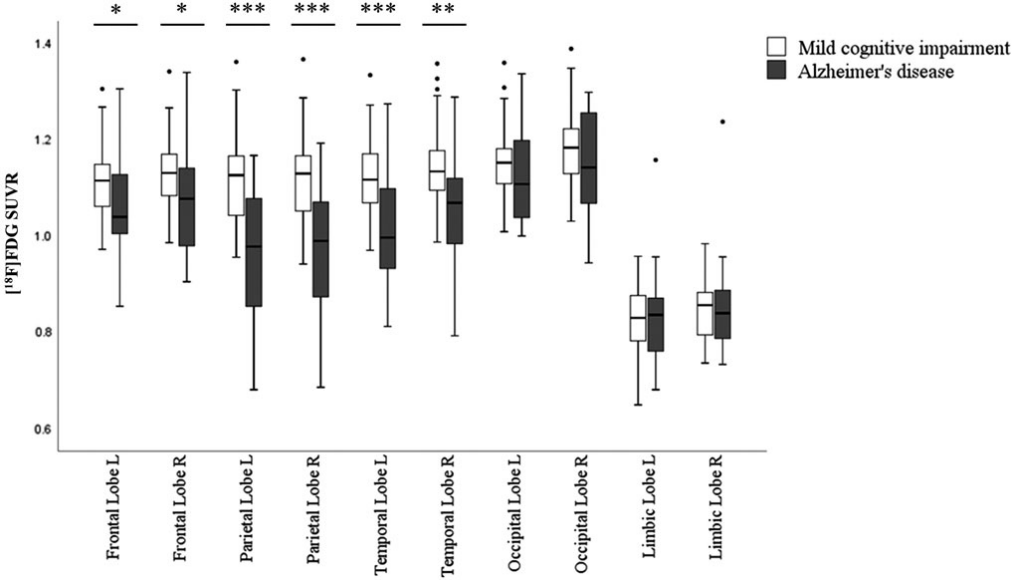
Box-whisker plot of differences in [^18^F]FDG SUVR (y-axis) in five main brain lobes (x-axis) between MCI and AD patients. Diagnostic groups are presented by different bar colors and outliers are shown as circles. Mann–Whitney *U* test over the diagnostic groups; *p < 0.05, **p < 0.01, ***p < 0.001. AD, Alzheimer's disease; [^18^F]FDG, [^18^F]fluorodeoxyglucose; MCI, mild cognitive impairment; SUVR, standardized uptake value ratio.

[^18^F]FDG-PET uptake in the brain was significantly different between clinically diagnosed MCI and AD patients in bilateral frontal (left *p* = 0.010, *U* = 333, right *p* = 0.017, *U* = 348), parietal (left *p* < 0.001, *U* = 202, right *p* < 0.001, *U* = 189), and temporal lobes (left *p* < 0.001, *U* = 244, right *p* = 0.001, *U* = 264) (Fig. 1). Consistent with the literature (Mosconi, 2005), the most pronounced difference in brain glucose metabolism was observed in temporoparietal lobes (*p* ≤ 0.001) in the present patient cohort. In line with our hypothesis, we have consequently focused on investigating functional connectivity alterations of the referred vulnerable brain regions (bilateral parietal and temporal lobes) in a correlative [^18^F]FDG-PET and EEG sLORETA analyses.

### Correlation between [^18^F]FDG-PET and EEG sLORETA connectivity measures in clinically diagnosed MCI and AD patients

In the first analysis, associations between brain glucose metabolism and sLORETA instantaneous and lagged linear connectivity in the corresponding temporal and parietal lobes were assessed in the whole cohort of clinically diagnosed MCI and AD patients (*n* = 67).

**FIG. 2.**
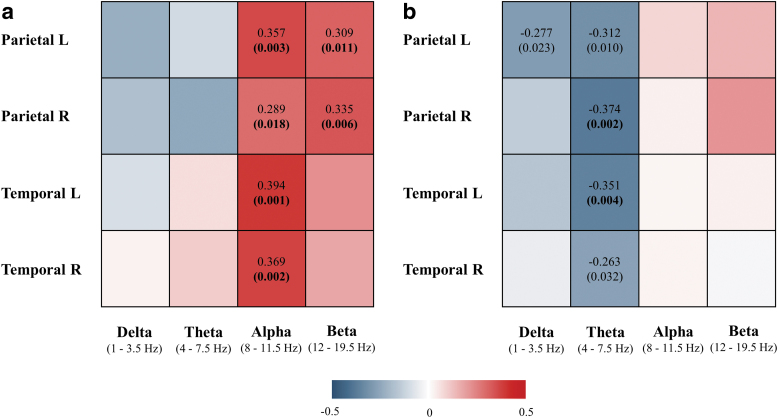
Correlation between brain [^18^F]FDG SUVR and sLORETA instantaneous **(a)** and lagged **(b)** linear connectivity in MCI and AD patients (*n* = 67). Colored areas represent the strength of the association (Spearman's correlation coefficient) between brain glucose metabolism and EEG measures of instantaneous and lagged linear connectivity in parietal L (left), parietal R (right), temporal L (left), and temporal R (right) lobes in four conventional frequency bands. Spearman's correlation coefficients (up) and *p*-values (below, in brackets) of significant associations (*p* < 0.05) are denoted on the plot, while *p*-values that remained significant after Benjamini–Hochberg correction for multiple comparisons are shown in bold. The red and blue colors indicate positive and negative correlations, respectively (as denoted on the color legend). EEG, electroencephalography; sLORETA, standardized low-resolution electromagnetic tomography.

Correlation analyses revealed significant associations between brain [^18^F]FDG SUVR and EEG instantaneous linear connectivity in alpha and beta frequency bands in parietal left (alpha: *r*_s_ = 0.357, *p* = 0.003; beta: *r*_s_ = 0.309, *p* = 0.011), parietal right (alpha: *r*_s_ = 0.289, *p* = 0.018; beta: *r*_s_ = 0.335, *p* = 0.006), temporal left (alpha: *r*_s_ = 0.394, *p* = 0.001), and temporal right lobes (alpha: *r*_s_ = 0.369, *p* = 0.002) (Fig. 2a, scatterplots presented in Supplementary Fig. S1). All correlations remained significant after correcting for multiple comparisons (Benjamini–Hochberg correction), and their *p*-values are shown in bold (Fig. 2a). In other words, decreased brain glucose metabolism in temporoparietal lobes correlated with decreased EEG instantaneous linear connectivity in fast frequency bands when analyzed in the same ROIs.

On the contrary, brain [^18^F]FDG SUVR was significantly associated with lagged linear connectivity in delta and theta frequency bands in parietal left (delta: *r*_s_ = −0.277, *p* = 0.023; theta: *r*_s_ = −0.312, *p* = 0.010), parietal right (theta: *r*_s_ = −0.374, *p* = 0.002), temporal left (theta: *r*_s_ = −0.351, *p* = 0.004), and temporal right lobes (theta: *r*_s_ = −0.263, *p* = 0.032) (Fig. 2b, scatterplots presented in Supplementary Fig. S2). Correlations in the right parietal and left temporal lobes remained significant after correction for multiple comparisons, and their *p*-values are shown in bold. Decreased brain glucose metabolism therefore correlated with increased EEG-lagged linear connectivity in slow frequency bands when analyzed in the same ROIs (temporoparietal lobes) (Fig. 2b).

To complete investigation of the spatial overlap between cortical glucose hypometabolism and altered brain functional connectivity, associations between [^18^F]FDG SUVR and sLORETA measures of instantaneous and lagged linear connectivity in four conventional frequency bands were additionally assessed in frontal and occipital lobes. The correlative analysis was not performed for the limbic lobe since sLORETA solution space was restricted to the cortical gray matter and, in contrast to the corresponding PET ROIs, did not include hippocampus. The measures of instantaneous and lagged linear connectivity were computed in the same manner; that is, they refer to the average connectivity of a localized region in relation to the rest of the functional network. In contrast to the temporal and parietal lobes, relationship between glucose uptake and EEG measures of functional connectivity in frontal and occipital lobes did not exhibit a consistent pattern of significant associations in clinically diagnosed MCI and AD patients nor in the subsample of CSF amyloid-positive patients (Supplementary Tables S1, S2, S3, S4).

### Correlation between [^18^F]FDG PET and EEG sLORETA connectivity measures in CSF amyloid-positive MCI and AD patients

In the second analysis, associations between brain glucose metabolism and EEG sLORETA connectivity measures were assessed in a group of amyloid-positive patients (*n* = 32, according to CSF Aβ42/40 ratio).

**FIG. 3.**
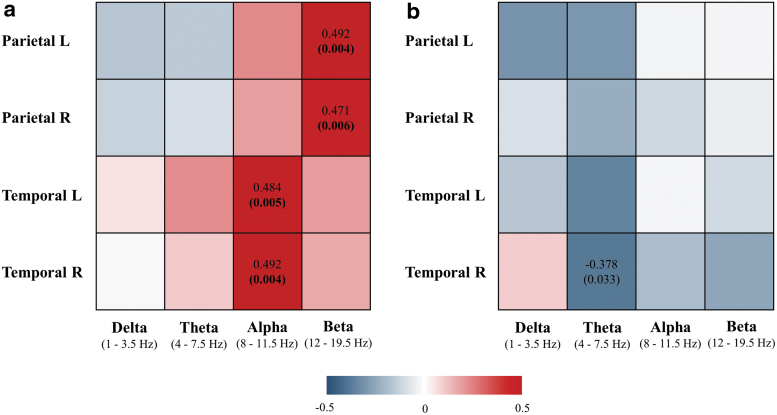
Correlation between brain [^18^F]FDG SUVR and sLORETA instantaneous **(a)** and lagged **(b)** linear connectivity in amyloid-positive MCI and AD patients (*n* = 32). Colored areas represent the strength of the association (Spearman's correlation coefficient) between brain glucose metabolism and EEG measures of instantaneous and lagged linear connectivity in parietal L (left), parietal R (right), temporal L (left), and temporal R (right) lobes in four conventional frequency bands. Spearman's correlation coefficients (up) and *p*-values (below, in brackets) of significant associations (*p* < 0.05) are denoted on the plot, while *p*-values that remained significant after Benjamini–Hochberg correction for multiple comparisons are shown in bold. The red and blue colors indicate positive and negative correlations, respectively (as denoted on the color legend).

Correlation analyses revealed significant association between brain [^18^F]FDG SUVR and EEG instantaneous linear connectivity in alpha and beta frequency bands in parietal left (beta: *r*_s_ = 0.492, *p* = 0.004), parietal right (beta: *r*_s_ = 0.471, *p* = 0.006), temporal left (alpha: *r*_s_ = 0.484, *p* = 0.005), and temporal right lobes (alpha: *r*_s_ = 0.492, *p* = 0.004), when analyzed in amyloid-positive patients only (Fig. 3a, scatterplots presented in Supplementary Fig. S3). All correlations remained significant after correcting for multiple comparisons (Benjamini–Hochberg correction), and their *p*-values are shown in bold (Fig. 3a). The pattern of observed relationships was similar as in the first analysis that included the entire cohort of MCI and AD patients; decreased glucose metabolism correlated with decreased EEG instantaneous linear connectivity in fast frequency bands in temporoparietal cortex.

On the contrary, brain [^18^F]FDG SUVR was significantly associated with EEG-lagged linear connectivity in the right temporal lobe only (theta band: *r*_s_ = −0.378, *p* = 0.033), when analyzed in amyloid-positive patients; however, it did not survive correction for multiple comparison (Fig. 3b, scatterplots presented in Supplementary Fig. S4).

There were no significant associations between [^18^F]FDG SUVRs and sLORETA instantaneous nor lagged linear connectivity in the temporal and parietal lobes when analyzed in the group of amyloid-negative patients (consisting of MCI patients, amyloid negative according to CSF Aβ42/40 ratio) (Supplementary Tables S5 and S6).

## Discussion

This study investigated relationship between topography of brain glucose metabolism and functional connectivity alterations as evidenced by [^18^F]FDG-PET and EEG sLORETA analysis, respectively, in patients along the AD continuum. Main findings of the study are as follows: brain glucose hypometabolism in temporoparietal lobes, core [^18^F]FDG-PET feature in AD, is associated with altered EEG functional connectivity and functional “disconnection” of the same cortical ROIs in clinically diagnosed MCI and AD patients and in a subsample of patients with biomarker evidence of AD pathology. The novelty of our study is that it demonstrated associations between glucose metabolism and EEG measures of both instantaneous and lagged linear connectivity across different frequency bands, providing comprehensive information on regional cortical disconnection associated with AD.

Cognitive processes are mediated by extensive functional connection and interaction between localized and specialized brain systems (Bressler and Menon, 2010). Therefore, localized neuropathology and consequent dysfunction of vulnerable brain areas, resembled by decreased metabolic (energetic demand) of the neurons, may lead to the “communication breakdown” among the affected brain regions. The patterns of associations between brain glucose hypometabolism and altered EEG connectivity measures in our study are similar when analyzed in patients with CSF-verified amyloid pathology compared with the group of patients clinically diagnosed with MCI and AD. Moreover, there were no significant associations between brain glucose metabolism and EEG connectivity measures in the group of amyloid-negative patients. The stratification was based on CSF Aβ42/40 ratio, known to correct for individual difference in amyloid production and to have a superior diagnostic performance compared with CSF Aβ42 levels alone (Hansson et al., 2007; Lewczuk et al., 2015). This aligns with the hypothesis of AD as a “disconnection syndrome” as the alterations in EEG functional connectivity within disease-affected brain regions are present in patients with the evidence of core AD molecular pathology, that is, along genuine AD continuum.

Both instantaneous and lagged synchronization of neuronal activity were shown to be physiologically important for inter- and intrahemispheric communication and function of memory systems in general (Fell and Axmacher, 2011; Gollo et al., 2011; Roelfsema et al., 1997). Instantaneous (zero-lag) synchronization between neuronal assemblies may emerge as a result of simultaneous input from another common brain region or through third relay station (Fell and Axmacher, 2011). Furthermore, it has been shown that zero-lag coupling can occur over distant brain regions due to the neuronal mechanisms that can compensate for the axonal delays (Traub et al., 1996; Bibbig et al., 2002). On the contrary, lagged synchronization presumably mirrors phase lag between neuronal activity due to the conductive latencies between brain regions (Fell and Axmacher, 2011). Our results further show that both of the referred measures of brain functional connectivity may provide information on brain network dysfunction due to AD pathology.

In more detail, brain glucose hypometabolism in left and right parietal and temporal lobes correlated with decreased EEG instantaneous linear connectivity in fast frequency bands (alpha and beta) when analyzed in the same ROIs in MCI and AD patients. Fast frequency neuronal synchronization has been shown to be implicated in executive and attentional functions as well as working and long-term memory (Fell and Axmacher, 2011; Palva and Palva, 2011). Numerous EEG measures of instantaneous connectivity derived from the surface EEG data, such as coherence and global field synchronization, have already shown to decrease in fast frequencies in cognitively impaired patients, correlate with cognitive scores and AD molecular markers (Smailovic and Jelic, 2019). Our EEG-PET imaging study further suggests localization, that is, temporoparietal cortex, and metabolic correlates, that is, association with glucose hypometabolism, of the reported decrease in EEG connectivity within fast frequencies. We emphasize that localized measures of EEG instantaneous dependence in both scalp and cortical source space should be interpreted with caution since they are confounded by volume conduction effect. However, they should not be completely disregarded from EEG studies. Physiological zero-lag synchrony between distant neuronal populations (cortical regions), as indicated above, has been confirmed in several studies (Fell and Axmacher, 2011; Traub et al., 1996; Vicente et al., 2008). Therefore, EEG investigations that exclusively employ and interpret lagged connectivity measures in the cortical source space overlook a significant portion of a physiologically meaningful zero-lag functional connectivity, whose disturbances in patients along AD continuum have already been thoroughly reported.

We further reported association between decreased brain glucose metabolism and increased EEG-lagged linear connectivity in slow frequency bands (delta and theta) in temporoparietal lobes in MCI and AD patients. Several EEG LORETA studies have described increase in lagged connectivity in slow frequency bands in patients along AD continuum. These alterations were most pronounced in temporal and frontal connections in theta (Canuet et al., 2012) and in widespread connections in delta band (Babiloni et al., 2018a). In addition, abnormal lagged connectivity in theta band was shown to correlate with cognitive decline, while increase in lagged connectivity in delta band differentiated well between AD, Parkinson's disease, and Lewy Body dementia patients (Babiloni et al., 2018a; Canuet et al., 2012). Concurrently, increase in resting-state EEG activity in slow frequencies is an abnormal feature and a characteristic EEG finding in cognitively impaired patients (Jelic and Kowalski, 2009). Even though the origin of delta and theta oscillations still remains speculative, several deep brain structures such as thalamus and limbic system including hippocampus, cingulate, and entorhinal cortex have been suggested as plausible contributors to the cortical slow frequency activity (Michel, 2009). The interpretation of EEG connectivity disturbances within slow frequency ranges might therefore involve both direct cortical ROIs and deep brain structures.

There are several proposed mechanisms behind increased functional connectivity in patients along AD continuum, including compensatory responses to the dysfunction of memory networks as well as synaptotoxicity and genuine neuronal failure due to the AD-related pathology (Canuet et al., 2012; Sperling et al., 2010). Furthermore, preclinical studies have pointed out that increased synchrony in cortical circuits in AD may be related to reduced GABAergic inhibition rather than increased excitatory transmission (Palop and Mucke, 2010). Since it was shown that activation of GABA pathways increases glucose metabolism in the brain (Peyron et al., 1994), we could further speculate that the association between reduced cortical glucose metabolism and increased functional connectivity might be a result of dysfunction of inhibitory neurons in AD.

Since EEG signals result from summated postsynaptic potentials and changes in ionic conductance across the neuronal membrane demand metabolic energy, a relationship between brain electrical activity and glucose metabolism was somewhat expected. In addition, the role of brain glucose hypometabolism, evidenced by [^18^F]FDG-PET, as a marker of synaptic function was substantiated by several studies. First, resting-state glucose metabolism was found to correlate with synaptophysin levels, presynaptic vesicle membrane protein, when analyzed in the corresponding brain regions (Rocher et al., 2003). Second, loss of synaptic density and activity leads to the decrease in neuronal energy demand and consequent reduction in the brain glucose metabolism (Mosconi, 2005). Third, recent studies provide evidence that [^18^F]FDG uptake is not a mere marker of brain neuronal activity as it reflects glucose uptake in astrocytes, important for synaptic function and plasticity (Zimmer et al., 2017). The latter notion might contribute to the part of variance not explained by the correlations between cortical glucose metabolism and EEG measures. Differences in methodological aspects of these techniques, such as spatial and temporal resolutions, could further rationalize moderate correlations.

The main limitations of this study are the cross-sectional design and a limited number of study participants. Even though the association between [^18^F]FDG-PET and EEG connectivity measures was not detected in the CSF amyloid-negative group, it should be further investigated since the group consisted of 10 patients only. Furthermore, large-scale multicentric studies with longitudinal design would allow for stratification of MCI patients on progressive (progressing to AD) and stabile MCI patients and evaluation of diagnostic and prognostic potential of EEG LORETA measures of brain functional connectivity in the context of AD.

## Conclusions

Our study demonstrated that topographical EEG measures of functional connectivity may detect regional dysfunction of AD-vulnerable brain areas as evidenced by association and spatial overlap with the cortical glucose hypometabolism in clinically diagnosed MCI and AD patients and in patients with biomarker-verified AD pathology. Moreover, our results indicate that EEG measures of both instantaneous and lagged linear connectivity might provide comprehensive information on regional cortical disconnection associated with AD. EEG is a noninvasive and widely accessible diagnostic technique that could have broad implication in clinical practice and AD drug trials. Future large-scale longitudinal studies are required to assess diagnostic and prognostic potential of topographical EEG measures of brain functional connectivity in patients along AD continuum.

## Authors' Contributions

U.S. contributed to the study design, data acquisition, data analyses, interpretation of results, and writing of article; T.K. contributed to the study design, data analyses, interpretation of results, and critical review and revision of the article; I.S. contributed to the data analyses, interpretation of results, and critical review and revision of the article; K.C. contributed to the data acquisition, interpretation of results, and critical review and revision of the article; A.N. contributed to the interpretation of results, and critical review and revision of the article; K.B. contributed to the data analyses, interpretation of results, and critical review and revision of the article; B.W. contributed to the interpretation of results and critical review and revision of the article; V.J. contributed to the study design, data acquisition, data analyses, interpretation of results, and critical review and revision of the article.

## Supplementary Material

Supplemental data

Supplemental data

Supplemental data

Supplemental data

Supplemental data

Supplemental data

Supplemental data

Supplemental data

Supplemental data

Supplemental data

## References

[B1] Babiloni C, Del Percio C, Caroli A, Salvatore E, Nicolai E, Marzano N, et al. 2016 Cortical sources of resting-state EEG rhythms are related to brain hypometabolism in subjects with Alzheimer's disease: an EEG-PET study. Neurobiol Aging 48:122–134 27668356 10.1016/j.neurobiolaging.2016.08.021

[B2] Babiloni C, Del Percio C, Lizio R, Noce G, Lopez S, Soricelli A, et al. 2018a Abnormalities of resting-state functional cortical connectivity in patients with dementia due to Alzheimer's and Lewy body diseases: an EEG study. Neurobiol Aging 65:18–40 29407464 10.1016/j.neurobiolaging.2017.12.023

[B3] Babiloni C, Del Percio C, Lizio R, Noce G, Lopez S, Soricelli A, et al. 2018b Functional cortical source connectivity of resting state electroencephalographic alpha rhythms shows similar abnormalities in patients with mild cognitive impairment due to Alzheimer's and Parkinson's diseases. Clin Neurophysiol 129:766–782 29448151 10.1016/j.clinph.2018.01.009

[B4] Bibbig A, Traub RD, Whittington MA 2002 Long-range synchronization of γ and β oscillations and the plasticity of excitatory and inhibitory synapses: A network model. J Neurophysiol 88:1634–1654 12364494 10.1152/jn.2002.88.4.1634

[B5] Bressler SL, Menon V 2010 Large-scale brain networks in cognition: emerging methods and principles. Trends Cogn Sci 14:277–290 20493761 10.1016/j.tics.2010.04.004

[B6] Brett M, Johnsrude IS, Owen AM 2002 The problem of functional localization in the human brain. Nat Rev Neurosci 3:243–249 11994756 10.1038/nrn756

[B7] Canuet L, Tellado I, Couceiro V, Fraile C, Fernandez-Novoa L, Ishii R, et al. 2012 Resting-state network disruption and APOE genotype in Alzheimer's disease: a lagged functional connectivity study. PLoS One 7:e46289-e 23050006 10.1371/journal.pone.0046289 PMC3457973

[B8] Chetelat G, Desgranges B, de la Sayette V, Viader F, Eustache F, Baron JC 2003 Mild cognitive impairment: can FDG-PET predict who is to rapidly convert to Alzheimer's disease? Neurology 60:1374–1377 12707450 10.1212/01.wnl.0000055847.17752.e6

[B9] Corder EH, Woodbury MA, Volkmann I, Madsen DK, Bogdanovic N, Winblad B 2000 Density profiles of Alzheimer disease regional brain pathology for the Huddinge brain bank: pattern recognition emulates and expands upon Braak staging. Exp Gerontol 35:851–864 11053676 10.1016/s0531-5565(00)00147-9

[B10] Delbeuck X, Van der Linden M, Collette F 2003 Alzheimer's disease as a disconnection syndrome? Neuropsychol Rev 13:79–92 12887040 10.1023/a:1023832305702

[B11] Desgranges B, Baron JC, de la Sayette V, Petit-Taboue MC, Benali K, Landeau B, et al. 1998 The neural substrates of memory systems impairment in Alzheimer's disease. A PET study of resting brain glucose utilization. Brain 121(Pt 4):611–631 9577389 10.1093/brain/121.4.611

[B12] Dierks T, Jelic V, Pascual-Marqui RD, Wahlund L, Julin P, Linden DE, et al. 2000 Spatial pattern of cerebral glucose metabolism (PET) correlates with localization of intracerebral EEG-generators in Alzheimer's disease. Clin Neurophysiol 111:1817–1824 11018498 10.1016/s1388-2457(00)00427-2

[B13] Engels MM, Stam CJ, van der Flier WM, Scheltens P, de Waal H, van Straaten EC 2015 Declining functional connectivity and changing hub locations in Alzheimer's disease: an EEG study. BMC Neurol 15:145 26289045 10.1186/s12883-015-0400-7 PMC4545875

[B14] Fell J, Axmacher N 2011 The role of phase synchronization in memory processes. Nat Rev Neurosci 12:105–118 21248789 10.1038/nrn2979

[B15] Foresta F, Morabito F, Marino S, Dattola S 2019 High-density EEG signal processing based on active-source reconstruction for brain network analysis in Alzheimer's disease. Electronics 8:1031

[B16] Gollo LL, Mirasso CR, Atienza M, Crespo-Garcia M, Cantero JL 2011 Theta band zero-lag long-range cortical synchronization via hippocampal dynamical relaying. PLoS One 6:e17756 21408082 10.1371/journal.pone.0017756 PMC3050931

[B17] Hansson O, Zetterberg H, Buchhave P, Andreasson U, Londos E, Minthon L, et al. 2007 Prediction of Alzheimer's disease using the CSF Abeta42/Abeta40 ratio in patients with mild cognitive impairment. Dement Geriatr Cogn Disord 23:316–320 17374949 10.1159/000100926

[B18] Herholz K, Salmon E, Perani D, Baron JC, Holthoff V, Frolich L, et al. 2002 Discrimination between Alzheimer dementia and controls by automated analysis of multicenter FDG PET. Neuroimage 17:302–316 12482085 10.1006/nimg.2002.1208

[B19] Jelic V, Kowalski J 2009 Evidence-based evaluation of diagnostic accuracy of resting EEG in dementia and mild cognitive impairment. Clin EEG Neurosci 40:129–142 19534305 10.1177/155005940904000211

[B20] Koenig T, Prichep L, Dierks T, Hubl D, Wahlund LO, John ER, et al. 2005 Decreased EEG synchronization in Alzheimer's disease and mild cognitive impairment. Neurobiol Aging 26:165–171 15582746 10.1016/j.neurobiolaging.2004.03.008

[B21] Leuzy A, Savitcheva I, Chiotis K, Lilja J, Andersen P, Bogdanovic N, et al. 2019 Clinical impact of [(18)F]flutemetamol PET among memory clinic patients with an unclear diagnosis. Eur J Nucl Med Mol Imaging 46:1276–1286 30915522 10.1007/s00259-019-04297-5 PMC6486908

[B22] Lewczuk P, Lelental N, Spitzer P, Maler JM, Kornhuber J 2015 Amyloid-beta 42/40 cerebrospinal fluid concentration ratio in the diagnostics of Alzheimer's disease: validation of two novel assays. J Alzheimers Dis 43:183–191 25079805 10.3233/JAD-140771

[B23] Ma CC, Liu AJ, Liu AH, Zhou XY, Zhou SN 2014 Electroencephalogram global field synchronization analysis: a new method for assessing the progress of cognitive decline in Alzheimer's disease. Clin EEG Neurosci 45:98–103 23986293 10.1177/1550059413489669

[B24] Mazziotta J, Toga A, Evans A, Fox P, Lancaster J, Zilles K, et al. 2001 A probabilistic atlas and reference system for the human brain: International Consortium for Brain Mapping (ICBM). Philos Trans R Soc Lond B Biol Sci 356:1293–1322 11545704 10.1098/rstb.2001.0915 PMC1088516

[B25] Michel CM 2009 Electrical Neuroimaging. Cambridge: Cambridge University Press, p. 238

[B26] Mosconi L 2005 Brain glucose metabolism in the early and specific diagnosis of Alzheimer's disease. Eur J Nucl Med Mol Imaging 32:486–510 15747152 10.1007/s00259-005-1762-7

[B27] Mulert C, Jager L, Schmitt R, Bussfeld P, Pogarell O, Moller HJ, et al. 2004 Integration of fMRI and simultaneous EEG: towards a comprehensive understanding of localization and time-course of brain activity in target detection. Neuroimage 22:83–94 15109999 10.1016/j.neuroimage.2003.10.051

[B28] Pagani M, Giuliani A, Oberg J, De Carli F, Morbelli S, Girtler N, et al. 2017a Progressive disintegration of brain networking from normal aging to Alzheimer disease: analysis of independent components of (18)F-FDG PET data. J Nucl Med 58:1132–1139 28280223 10.2967/jnumed.116.184309

[B29] Pagani M, Nobili F, Morbelli S, Arnaldi D, Giuliani A, Oberg J, et al. 2017b Early identification of MCI converting to AD: a FDG PET study. Eur J Nucl Med Mol Imaging 44:2042–2052 28664464 10.1007/s00259-017-3761-x

[B30] Palmqvist S, Schöll M, Strandberg O, Mattsson N, Stomrud E, Zetterberg H, et al. 2017 Earliest accumulation of β-amyloid occurs within the default-mode network and concurrently affects brain connectivity. Nat Commun 8:1214 29089479 10.1038/s41467-017-01150-x PMC5663717

[B31] Palop JJ, Mucke L 2010 Amyloid-β induced neuronal dysfunction in Alzheimer's disease: from synapses toward neural networks. Nat Neurosci 13:812–818 20581818 10.1038/nn.2583 PMC3072750

[B32] Palva S, Palva JM 2011 Functional roles of alpha-band phase synchronization in local and large-scale cortical networks. Front Psychol 2:204 21922012 10.3389/fpsyg.2011.00204 PMC3166799

[B33] Pascual-Marqui RD 1999 Review of methods for solving the EEG inverse problem. Conf Proc 1:77–90

[B34] Pascual-Marqui RD 2002 Standardized low-resolution brain electromagnetic tomography (sLORETA): technical details. Methods Find Exp Clin Pharmacol 24(Suppl D):5–12 12575463

[B35] Pascual-Marqui RD 2007a Discrete, 3D distributed, linear imaging methods of electric neuronal activity. Part 1: exact, zero error localization. arXiv 0710.3341.

[B36] Pascual-Marqui RD 2007b Instantaneous and lagged measurements of linear and nonlinear dependence between groups of multivariate time series: frequency decomposition. arXiv 07111455

[B37] Pascual-Marqui RD, Lehmann D, Koukkou M, Kochi K, Anderer P, Saletu B, et al. 2011 Assessing interactions in the brain with exact low-resolution electromagnetic tomography. Philos Trans A Math Phys Eng Sci 369:3768–3784 21893527 10.1098/rsta.2011.0081

[B38] Pascual-Marqui RD, Michel CM, Lehmann D 1994 Low resolution electromagnetic tomography: a new method for localizing electrical activity in the brain. Int J Psychophysiol 18:49–65 7876038 10.1016/0167-8760(84)90014-x

[B39] Peyron R, Le Bars D, Cinotti L, Garcia-Larrea L, Galy G, Landais P, et al. 1994 Effects of GABAA receptors activation on brain glucose metabolism in normal subjects and temporal lobe epilepsy (TLE) patients. A positron emission tomography (PET) study. Part I: brain glucose metabolism is increased after GABAA receptors activation. Epilepsy Res 19:45–54 7813413 10.1016/0920-1211(94)90087-6

[B40] Rocher AB, Chapon F, Blaizot X, Baron JC, Chavoix C 2003 Resting-state brain glucose utilization as measured by PET is directly related to regional synaptophysin levels: a study in baboons. Neuroimage 20:1894–1898 14642499 10.1016/j.neuroimage.2003.07.002

[B41] Roelfsema PR, Engel AK, König P, Singer W 1997 Visuomotor integration is associated with zero time-lag synchronization among cortical areas. Nature 385:157–161 8990118 10.1038/385157a0

[B42] Selkoe DJ, Hardy J 2016 The amyloid hypothesis of Alzheimer's disease at 25 years. EMBO Mol Med 8:595–608 27025652 10.15252/emmm.201606210 PMC4888851

[B43] Smailovic U, Jelic V 2019 Neurophysiological markers of Alzheimer's disease: quantitative EEG approach. Neurol Ther 8(Suppl 2):37–55 31833023 10.1007/s40120-019-00169-0 PMC6908537

[B44] Smailovic U, Koenig T, Kareholt I, Andersson T, Kramberger MG, Winblad B, Jelic V 2018 Quantitative EEG power and synchronization correlate with Alzheimer's disease CSF biomarkers. Neurobiol Aging 63:88–95 29245058 10.1016/j.neurobiolaging.2017.11.005

[B45] Sperling RA, Aisen PS, Beckett LA, Bennett DA, Craft S, Fagan AM, et al. 2011 Toward defining the preclinical stages of Alzheimer's disease: recommendations from the National Institute on Aging-Alzheimer's Association workgroups on diagnostic guidelines for Alzheimer's disease. Alzheimers Dement 7:280–292 21514248 10.1016/j.jalz.2011.03.003 PMC3220946

[B46] Sperling RA, Dickerson BC, Pihlajamaki M, Vannini P, LaViolette PS, Vitolo OV, et al. 2010 Functional alterations in memory networks in early Alzheimer's disease. Neuromolecular Med 12:27–43 20069392 10.1007/s12017-009-8109-7 PMC3036844

[B47] Stam CJ, Nolte G, Daffertshofer A 2007 Phase lag index: assessment of functional connectivity from multi channel EEG and MEG with diminished bias from common sources. Hum Brain Mapp 28:1178–1193 17266107 10.1002/hbm.20346 PMC6871367

[B48] Terry RD, Masliah E, Salmon DP, Butters N, DeTeresa R, Hill R, et al. 1991 Physical basis of cognitive alterations in Alzheimer's disease: synapse loss is the major correlate of cognitive impairment. Ann Neurol 30:572–580 1789684 10.1002/ana.410300410

[B49] Toussaint P-J, Perlbarg V, Bellec P, Desarnaud S, Lacomblez L, Doyon J, et al. 2012 Resting state FDG-PET functional connectivity as an early biomarker of Alzheimer's disease using conjoint univariate and independent component analyses. Neuroimage 63:936–946 22510256 10.1016/j.neuroimage.2012.03.091

[B50] Traub RD, Whittington MA, Stanford IM, Jefferys JGR 1996 A mechanism for generation of long-range synchronous fast oscillations in the cortex. Nature 383:621–624 8857537 10.1038/383621a0

[B51] Tzourio-Mazoyer N, Landeau B, Papathanassiou D, Crivello F, Etard O, Delcroix N, et al. 2002 Automated anatomical labeling of activations in SPM using a macroscopic anatomical parcellation of the MNI MRI single-subject brain. Neuroimage 15:273–289 11771995 10.1006/nimg.2001.0978

[B52] Uylings HB, de Brabander JM 2002 Neuronal changes in normal human aging and Alzheimer's disease. Brain Cogn 49:268–276 12139954 10.1006/brcg.2001.1500

[B53] Vicente R, Gollo LL, Mirasso CR, Fischer I, Pipa G 2008 Dynamical relaying can yield zero time lag neuronal synchrony despite long conduction delays. Proc Natl Acad Sci U S A 105:17157 18957544 10.1073/pnas.0809353105 PMC2575223

[B54] Winblad B, Palmer K, Kivipelto M, Jelic V, Fratiglioni L, Wahlund LO, et al. 2004 Mild cognitive impairment—beyond controversies, towards a consensus: report of the International Working Group on Mild Cognitive Impairment. J Intern Med 256:240–246 15324367 10.1111/j.1365-2796.2004.01380.x

[B55] World Health Organization. 1992 The ICD-10 Classification of Mental and Behavioural Disorders: Clinical Descriptions and Diagnostic Guidelines. Geneva: WHO

[B56] Worrell GA, Lagerlund TD, Sharbrough FW, Brinkmann BH, Busacker NE, Cicora KM, et al. 2000 Localization of the epileptic focus by low-resolution electromagnetic tomography in patients with a lesion demonstrated by MRI. Brain Topogr 12:273–282 10912735 10.1023/a:1023407521772

[B57] Zimmer ER, Parent MJ, Souza DG, Leuzy A, Lecrux C, Kim H-I, et al. 2017 [18F]FDG PET signal is driven by astroglial glutamate transport. Nat Neurosci 20:393 28135241 10.1038/nn.4492 PMC5378483

[B58] Zumsteg D, Friedman A, Wieser HG, Wennberg RA 2006 Propagation of interictal discharges in temporal lobe epilepsy: correlation of spatiotemporal mapping with intracranial foramen ovale electrode recordings. Clin Neurophysiol 117:2615–2626 17029950 10.1016/j.clinph.2006.07.319

