# Supplementary material for: Regional Disconnection in Alzheimer Dementia and Amyloid-Positive Mild Cognitive Impairment: Association Between EEG Functional Connectivity and Brain Glucose Metabolism
Source: Brain Connect. 2020 Dec 14;10(10):555–65. doi: 10.1089/brain.2020.0785 (PMC7757561; doi:10.1089/brain.2020.0785)
Supplement: Supplemental data [file Supp_TableS1.docx]

**Supplementary Table 1.** Correlation between brain [^18^F]FDG SUVR and sLORETA instantaneous linear connectivity in frontal L (left), frontal R (right), occipital L (left) and occipital R (right) lobes in four conventional frequency bands in MCI and AD patients.

|  | **Delta** | **Theta** | **Alpha** | **Beta** |
| --- | --- | --- | --- | --- |
| **Frontal L** | r_s_ = -0.028 | r_s_ = 0.048 | r_s_ = 0.154 | r_s_ = 0.070 |
|  | (p = 0.819) | (p = 0.701) | (p = 0.213) | (p = 0.576) |
| **Frontal R** | r_s_ = -0.028 | r_s_ = 0.121 | r_s_ = 0.246 | r_s_ = 0.126 |
|  | (p = 0.822) | (p = 0.331) | (p = 0.045) | (p = 0.311) |
| **Occipital L** | r_s_ = -0.166 | r_s_ = -0.006 | r_s_ = 0.210 | r_s_ = 0.084 |
|  | (p = 0.180) | (p = 0.961) | (p = 0.089) | (p = 0.499) |
| **Occipital R** | r_s_ = -0.006 | r_s_ = 0.025 | r_s_ = 0.175 | r_s_ = -0.039 |
|  | (p = 0.963) | (p = 0.843) | (p = 0.156) | (p = 0.757) |

Results are presented as correlations between brain glucose metabolism ([^18^F]FDG SUVR) and EEG instantaneous linear connectivity measures within each ROI and in four conventional frequency bands in all MCI and AD patients (n = 67). Spearman's correlation coefficients (r_s_) and p-values.
