# Supplementary material for: Regional Disconnection in Alzheimer Dementia and Amyloid-Positive Mild Cognitive Impairment: Association Between EEG Functional Connectivity and Brain Glucose Metabolism
Source: Brain Connect. 2020 Dec 14;10(10):555–65. doi: 10.1089/brain.2020.0785 (PMC7757561; doi:10.1089/brain.2020.0785)
Supplement: Supplemental data [file Supp_TableS2.docx]

**Supplementary Table 2.** Correlation between brain [^18^F]FDG SUVR and sLORETA lagged linear connectivity in frontal L (left), frontal R (right), occipital L (left) and occipital R (right) lobes in four conventional frequency bands in MCI and AD patients.

|  | **Delta** | **Theta** | **Alpha** | **Beta** |
| --- | --- | --- | --- | --- |
| **Frontal L** | r_s_ = -0.125 | r_s_ = -0.114 | r_s_ = -0.041 | r_s_ = 0.169 |
|  | (p = 0.313) | (p = 0.360) | (p = 0.742) | (p = 0.171) |
| **Frontal R** | r_s_ = -0.156 | r_s_ = -0.157 | r_s_ = -0.019 | r_s_ = 0.284 |
|  | (p = 0.208) | (p = 0.206) | (p = 0.877) | (p = 0.020) |
| **Occipital L** | r_s_ = -0.161 | r_s_ = -0.233 | r_s_ = 0.043 | r_s_ = -0.065 |
|  | (p = 0.194) | (p = 0.058) | (p = 0.728) | (p = 0.601) |
| **Occipital R** | r_s_ = -0.063 | r_s_ = -0.165 | r_s_ = 0.062 | r_s_ = -0.127 |
|  | (p = 0.614) | (p = 0.183) | (p = 0.619) | (p = 0.304) |

Results are presented as correlations between brain glucose metabolism ([^18^F]FDG SUVR) and EEG lagged linear connectivity measures within each ROI and in four conventional frequency bands in all MCI and AD patients (n = 67). Spearman's correlation coefficients (r_s_) and p-values.
