# Supplementary material for: Regional Disconnection in Alzheimer Dementia and Amyloid-Positive Mild Cognitive Impairment: Association Between EEG Functional Connectivity and Brain Glucose Metabolism
Source: Brain Connect. 2020 Dec 14;10(10):555–65. doi: 10.1089/brain.2020.0785 (PMC7757561; doi:10.1089/brain.2020.0785)
Supplement: Supplemental data [file Supp_TableS3.docx]

**Supplementary Table 3.** Correlation between brain [^18^F]FDG SUVR and sLORETA instantaneous linear connectivity in frontal L (left), frontal R (right), occipital L (left) and occipital R (right) lobes in four conventional frequency bands in amyloid positive MCI and AD patients.

|  | **Delta** | **Theta** | **Alpha** | **Beta** |
| --- | --- | --- | --- | --- |
| **Frontal L** | r_s_ = 0.208 | r_s_ = 0.113 | r_s_ = 0.123 | r_s_ = 0.149 |
|  | (p = 0.253) | (p = 0.540) | (p = 0.503) | (p = 0.415) |
| **Frontal R** | r_s_ = 0.173 | r_s_ = 0.146 | r_s_ = 0.207 | r_s_ = 0.195 |
|  | (p = 0.345) | (p = 0.424) | (p = 0.256) | (p = 0.285) |
| **Occipital L** | r_s_ = -0.060 | r_s_ = 0.018 | r_s_ = 0.111 | r_s_ = 0.222 |
|  | (p = 0.745) | (p = 0.924) | (p = 0.544) | (p = 0.223) |
| **Occipital R** | r_s_ = -0.029 | r_s_ = 0.077 | r_s_ = 0.181 | r_s_ = 0.084 |
|  | (p = 0.875) | (p = 0.674) | (p = 0.321) | (p = 0.646) |

Results are presented as correlations between brain glucose metabolism ([^18^F]FDG SUVR) and EEG instantaneous linear connectivity measures within each ROI and in four conventional frequency bands in amyloid positive MCI and AD patients (n = 32). Spearman's correlation coefficients (r_s_) and p-values.
