# Supplementary material for: Regional Disconnection in Alzheimer Dementia and Amyloid-Positive Mild Cognitive Impairment: Association Between EEG Functional Connectivity and Brain Glucose Metabolism
Source: Brain Connect. 2020 Dec 14;10(10):555–65. doi: 10.1089/brain.2020.0785 (PMC7757561; doi:10.1089/brain.2020.0785)
Supplement: Supplemental data [file Supp_TableS4.docx]

**Supplementary Table 4.** Correlation between brain [^18^F]FDG SUVR and sLORETA lagged linear connectivity in frontal L (left), frontal R (right), occipital L (left) and occipital R (right) lobes in four conventional frequency bands in amyloid positive MCI and AD patients.

|  | **Delta** | **Theta** | **Alpha** | **Beta** |
| --- | --- | --- | --- | --- |
| **Frontal L** | r_s_ = -0.150 | r_s_ = -0.219 | r_s_ = -0.207 | r_s_ = 0.003 |
|  | (p = 0.413) | (p = 0.228) | (p = 0.256) | (p = 0.987) |
| **Frontal R** | r_s_ = -0.118 | r_s_ = -0.311 | r_s_ = -0.295 | r_s_ = 0.125 |
|  | (p = 0.519) | (p = 0.083) | (p = 0.101) | (p = 0.494) |
| **Occipital L** | r_s_ = 0.102 | r_s_ = -0.367 | r_s_ = -0.106 | r_s_ = -0.037 |
|  | (p = 0.579) | (p = 0.039) | (p = 0.564) | (p = 0.841) |
| **Occipital R** | r_s_ = 0.130 | r_s_ = -0.276 | r_s_ = -0.284 | r_s_ = -0.155 |
|  | (p = 0.479) | (p = 0.126) | (p = 0.115) | (p = 0.398) |

Results are presented as correlations between brain glucose metabolism ([^18^F]FDG SUVR) and EEG lagged linear connectivity measures within each ROI and in four conventional frequency bands in amyloid positive MCI and AD patients (n = 32). Spearman's correlation coefficients (r_s_) and p-values.
