# Supplementary material for: Regional Disconnection in Alzheimer Dementia and Amyloid-Positive Mild Cognitive Impairment: Association Between EEG Functional Connectivity and Brain Glucose Metabolism
Source: Brain Connect. 2020 Dec 14;10(10):555–65. doi: 10.1089/brain.2020.0785 (PMC7757561; doi:10.1089/brain.2020.0785)
Supplement: Supplemental data [file Supp_FigS4.docx]

**
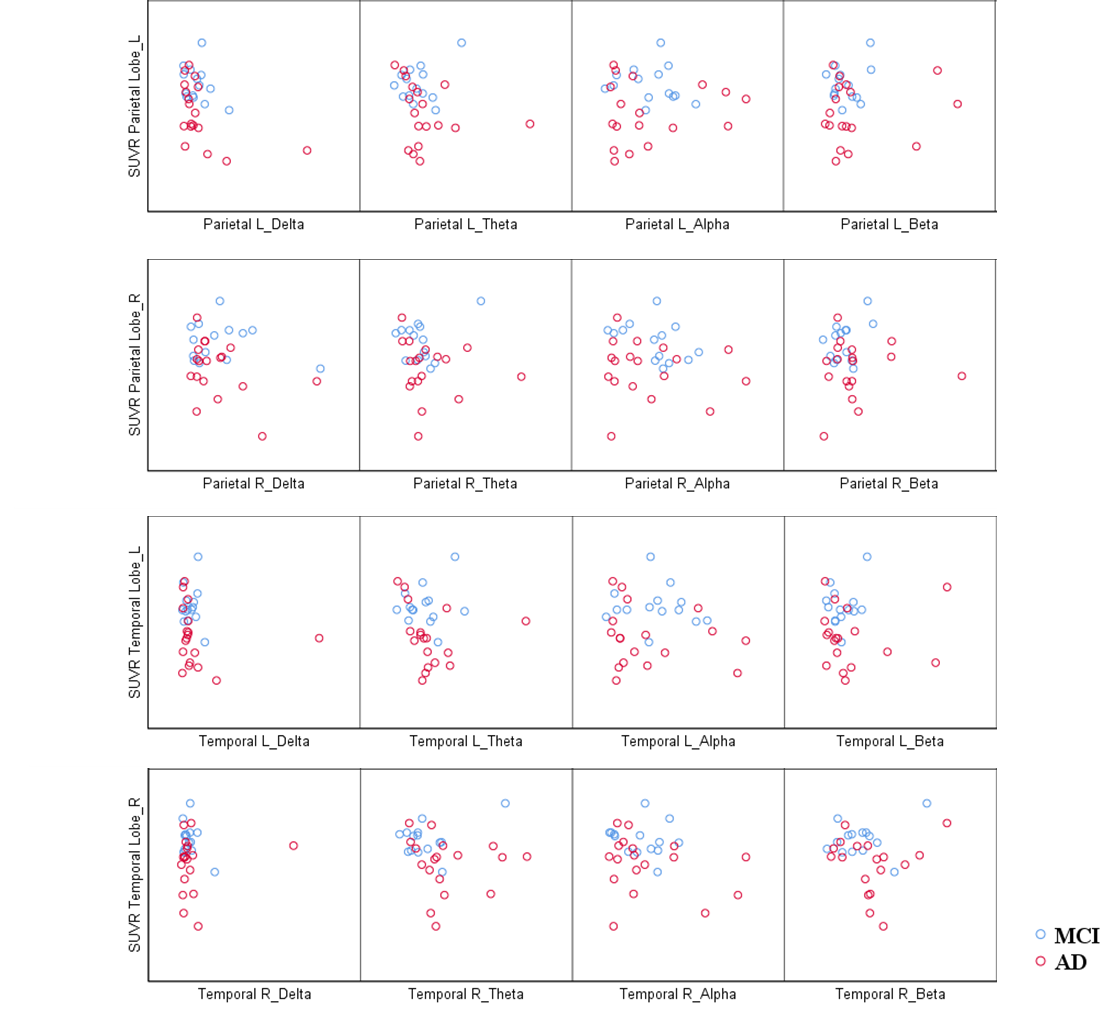
Supplementary Figure 4.** Relationship between brain [18F]FDG SUVR (y-axis) and sLORETA lagged linear connectivity (x-axis) in amyloid positive MCI and AD patients (n = 32). Data shown as a matrix of scatterplots with individual data points representing brain glucose metabolism and lagged linear connectivity in parietal L (left), parietal R (right), temporal L (left) and temporal R (right) lobes in four conventional frequency bands.
