# Supplementary material for: Regional Disconnection in Alzheimer Dementia and Amyloid-Positive Mild Cognitive Impairment: Association Between EEG Functional Connectivity and Brain Glucose Metabolism
Source: Brain Connect. 2020 Dec 14;10(10):555–65. doi: 10.1089/brain.2020.0785 (PMC7757561; doi:10.1089/brain.2020.0785)
Supplement: Supplemental data [file Supp_TableS5.docx]

**Supplementary Table 5.** Correlation between brain [^18^F]FDG SUVR and sLORETA instantaneous linear connectivity in temporoparietal lobes in amyloid negative MCI patients.

|  | **Delta** | **Theta** | **Alpha** | **Beta** |
| --- | --- | --- | --- | --- |
| **Parietal L** | r_s_ = 0.006  (p = 0.987) | r_s_ = -0.127  (p = 0.726) | r_s_ = 0.055  (p = 0.881) | r_s_ = -0.418  (p = 0.229) |
| **Parietal R** | r_s_ = -0.055  (p = 0.881) | r_s_ = -0.273  (p = 0.446) | r_s_ = -0.103  (p = 0.777) | r_s_ = -0.418  (p = 0.229) |
| **Temporal L** | r_s_ = 0.055  (p = 0.881) | r_s_ = 0.442  (p = 0.200) | r_s_ = 0.394  (p = 0.260) | r_s_ = -0.103  (p = 0.777) |
| **Temporal R** | r_s_ = 0.491  (p = 0.150) | r_s_ = 0.467  (p = 0.174) | r_s_ = 0.576  (p = 0.082) | r_s_ = 0.321  (p = 0.365) |

Results are presented as correlations between brain glucose metabolism ([^18^F]FDG SUVR) and EEG instantaneous linear connectivity measures within each ROI and in four conventional frequency bands in CSF amyloid negative (according to CSF Aβ42/40 ratio; cutoff < 0.89) MCI patients (n = 10). Spearman's correlation coefficients (r_s_) and p-values.
