# Supplementary material for: Regional Disconnection in Alzheimer Dementia and Amyloid-Positive Mild Cognitive Impairment: Association Between EEG Functional Connectivity and Brain Glucose Metabolism
Source: Brain Connect. 2020 Dec 14;10(10):555–65. doi: 10.1089/brain.2020.0785 (PMC7757561; doi:10.1089/brain.2020.0785)
Supplement: Supplemental data [file Supp_TableS6.docx]

**Supplementary Table 6.** Correlation between brain [^18^F]FDG SUVR and sLORETA lagged linear connectivity in temporoparietal lobes in amyloid negative MCI patients.

|  | **Delta** | **Theta** | **Alpha** | **Beta** |
| --- | --- | --- | --- | --- |
| **Parietal L** | r_s_ = 0.224  (p = 0.533) | r_s_ = -0.115  (p = 0.751) | r_s_ = -0.055  (p = 0.881) | r_s_ = 0.018  (p = 0.960) |
| **Parietal R** | r_s_ = 0.139  (p = 0.701) | r_s_ = -0.115  (p = 0.751) | r_s_ = 0.115  (p = 0.751) | r_s_ = 0.455  (p = 0.187) |
| **Temporal L** | r_s_ = 0.091  (p = 0.803) | r_s_ = -0.539  (p = 0.108) | r_s_ = -0.236  (p = 0.511) | r_s_ = -0.188  (p = 0.603) |
| **Temporal R** | r_s_ = -0.079  (p = 0.829) | r_s_ = 0.067  (p = 0.855) | r_s_ = 0.309  (p = 0.385) | r_s_ = -0.515  (p = 0.128) |

Results are presented as correlations between brain glucose metabolism ([^18^F]FDG SUVR) and EEG lagged linear connectivity measures within each ROI and in four conventional frequency bands in CSF amyloid negative (according to CSF Aβ42/40 ratio; cutoff < 0.89) MCI patients (n = 10). Spearman's correlation coefficients (r_s_) and p-values.
